# Role of interleukins in the pathogenesis of pulmonary fibrosis

**DOI:** 10.1038/s41420-021-00437-9

**Published:** 2021-03-15

**Authors:** Yi Xin She, Qing Yang Yu, Xiao Xiao Tang

**Affiliations:** State Key Laboratory of Respiratory Disease, National Clinical Research Center for Respiratory Disease, National Center for Respiratory Medicine, Guangzhou Institute of Respiratory Health, The First Affiliated Hospital of Guangzhou Medical University, Guangzhou, China

**Keywords:** Pathogenesis, Cell biology

## Abstract

Interleukins, a group of cytokines participating in inflammation and immune response, are proved to be involved in the formation and development of pulmonary fibrosis. In this article, we reviewed the relationship between interleukins and pulmonary fibrosis from the clinical, animal, as well as cellular levels, and discussed the underlying mechanisms in vivo and in vitro. Despite the effects of interleukin-targeted treatment on experimental pulmonary fibrosis, clinical applications are lacking and unsatisfactory. We conclude that intervening in one type of interleukins with similar functions in IPF may not be enough to stop the development of fibrosis as it involves a complex network of regulation mechanisms. Intervening interleukins combined with other existing therapy or targeting interleukins affecting multiple cells/with different functions at the same time may be one of the future directions. Furthermore, the intervention time is critical as some interleukins play different roles at different stages. Further elucidation on these aspects would provide new perspectives on both the pathogenesis mechanism, as well as the therapeutic strategy and drug development.

## Introduction

Interleukins (ILs) are a type of cytokines with immunoregulatory functions and derived from a variety of cells, including macrophages, T lymphocytes, mast cells, stromal cells, epithelial cells, and neutrophils, etc.^1,2^. In 1977, IL-1 was first discovered and at least 38 interleukins have been found thereafter. According to the sequence homology, as well as their main functions and receptors, interleukins are divided into IL-1 family, γc family, chemokine family, IL-10 family, IL-6/IL-12 family, and IL-17 family^1^. They are classified as type-1 (Th1-like) and type-2 (Th2-like) cytokines based on their immune responses as well^3^. Their functions are also complex and diverse. Generally speaking, interleukins regulate the immune system by participating in innate immune responses, promoting the proliferation and differentiation of immune cells, and specifically recruiting inflammatory cells^4–8^. In addition, some interleukins are also critical for inflammation responses and hematopoiesis^9–13^. A number of studies have evidenced that interleukins are associated with some autoimmune diseases (e.g., rheumatoid arthritis) and tumorigenesis^14^.

Pulmonary fibrosis (PF) is a chronic, progressive fibrotic lung pathological change that can be observed in idiopathic pulmonary fibrosis (IPF), systemic sclerosis, silicosis, and other lung diseases, characterized by damage to the alveolar structure and the replacement of normal lung tissue by deposited extracellular matrix, which resulted in respiratory failure and death^15,16^. The most common idiopathic interstitial pneumonia is IPF, which manifests as usual interstitial pneumonia (UIP) with temporal and spatial heterogeneity^17,18^. Incidence of IPF is estimated to range between 2 and 30 cases per 100,000 people per year, with a median survival of 2–3 years from diagnosis^19,20^. The treatment for IPF is very limited. Although Pirfenidone and Nintedanib have been recommended for clinical use, the efficacy is still insufficient to cure the disease^21^. Therefore, clarifying the pathogenesis of IPF is of great significance to therapeutic strategy.

Damage, aberrant senescence, and apoptosis of alveolar epithelial cells (AECs), as well as dysfunctional repair after injury leading to tissue fibrotic changes, is regarded as the core pathogenesis of IPF^18,22–26^. The immune response is also involved in the development of IPF^27,28^. Under the action of chemokines, circulating immune cells are recruited to lesions, where there are abnormal AECs and accumulated fibroblasts. Th1/Th2 imbalance promotes pulmonary fibrosis through pro-fibrotic factors and inflammatory cytokines^29^. Furthermore, immune regulatory mechanisms dominated by macrophages and/or dendritic cells (DCs) have also been documented in IPF^30–34^. These phenomena caused by immune cells seem to be closely associated with cytokines. A hypothesis called the “phagocytosis-secretion-immunity” network of macrophages explained the relationship between immune cells, cytokines, and pulmonary fibrosis^35^. Therefore, the unbalanced secretion of cytokines may be the key cause of aberrant cell function that results in immunologic derangement in pulmonary fibrosis.

## Interleukins and pulmonary fibrosis in clinical research

Interleukin levels in lung tissue, bronchoalveolar lavage fluid (BALF), or blood are altered in patients with pulmonary fibrosis (Table 1). IL-1β and IL-17A in BALF, as well as IL-2, IL-10, IL-12 in serum, were higher in IPF patients as compared to healthy subjects^36,37^. Interleukin levels not only change between subjects with and without pulmonary fibrosis, but also between different stages of pulmonary fibrosis. For example, peripheral blood levels of IL-6 and IL-9 increased in patients with acute exacerbation IPF (AE-IPF), compared with those with stable IPF^38,39^.

**Table 1:** Changes of interleukin levels in patients with pulmonary fibrosis.

Interleukin	Group	Sample	Method	Fold change	Ref.
IL-1β	IPF vs. HC	BALF	ELISA	↑, 2.23	^36^
IL-2	IPF vs. HC	Serum	ELISA	↑, 10	^37^
IL-6	AE-IPF vs. HC	Serum	Protein microarray analysis	↑, 1.36	^39^
	AE-IPF vs. stable IPF	Serum		↑, 1.19	^39^
IL-8	IPF vs. HC	Serum	ELISA	↑, 2.67	^37^
	IPF vs. HC	Serum and BALF	ELISA	↑, >10	^40^
IL-9	AE-IPF vs. stable IPF	Serum	Protein microarray analysis	↑, 1.50	^39^
IL-10	IPF vs. HC	Serum	ELISA	↑, 10.12	^37^
IL-12	IPF vs. HC	Serum	ELISA	↑, 6.92	^37^
IL-17A	IPF vs. HC	BALF	ELISA	↑, 9.67	^36^
IL-33	IPF vs. HC	BALF	ELISA	↑, 3.59	^42^

*AE-IPF* acute exacerbation-idiopathic pulmonary fibrosis, *BALF* bronchoalveolar lavage fluid, *HC* healthy control, *IPF* idiopathic pulmonary fibrosis.

Conversely, interleukin levels reflect the degree of inflammation and poor prognosis in pulmonary fibrosis and can be used to assess disease progression and severity. For instance, IL-8 (a member of the CXC chemokine family) level in serum reflects the degree of neutrophilic alveolitis in IPF, and its level in lung tissue is negatively correlated with lung function^37,40^. Moreover, high and low concentrations of IL-8 in plasma are correlated with a median survival of 1.9 and 5.1 years, respectively^41^. And mortality was increased by 6.7% for each 1 pg/mL in serum concentrations of IL-8^38,39^.

Disorders of interleukins might affect the formation and development of pulmonary fibrosis. IL-33 (a member of the IL-1 family) and thymic stromal lymphopoietin co-stimulate the upstream and downstream signals of IL-13 ^42^. An increased IL-13 level and its inducible proteins and factors (such as periostin and CCL2) in IPF may accelerate the process of pulmonary fibrosis by inhibiting epithelial wound healing^43^.

## Interleukins and pulmonary fibrosis in animal models

**Fig. 1:**
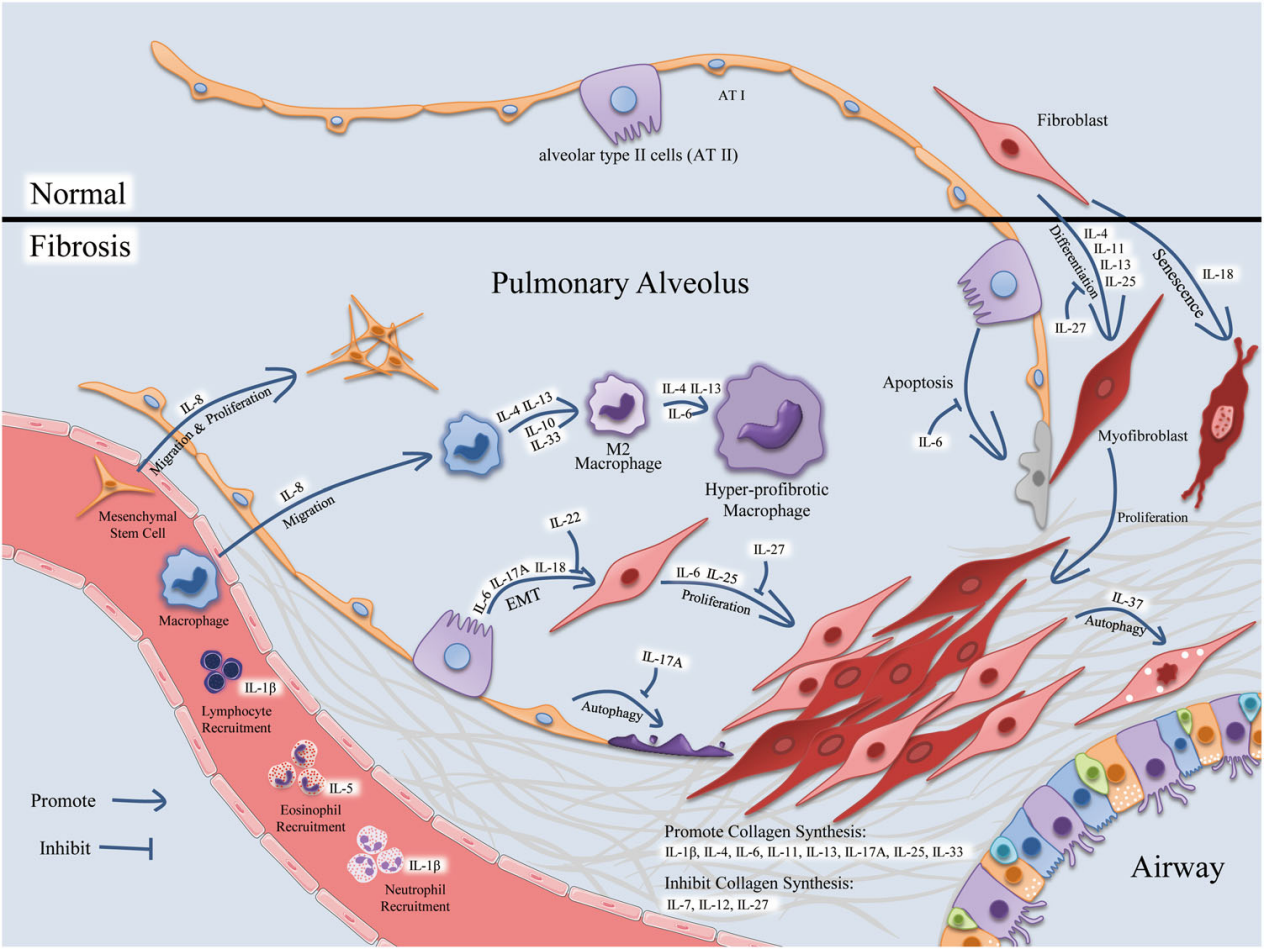
Interleukins affect the morphology and functions of various cells in pulmonary fibrosis. (1) Mesenchymal stem cells: IL-8 facilitates the migration and proliferation of mesenchymal stem cells. (2) Macrophages: IL-8 also induces migration of macrophages. IL-4, IL-13, IL-10, and IL-33 promote macrophages to transform into M2 phenotype, which is further promoted by IL-4, IL-6, and IL-13 to transform into a hyper-profibrotic phenotype. (3) Other immune cells: IL-1β induces recruitment of lymphocytes and neutrophils, while IL-5 induces recruitment of eosinophils. (4) Alveolar epithelial cells: IL-17A inhibits autophagy of alveolar epithelial cells, and IL-6 suppresses apoptosis of alveolar type II cells (AT II). IL-6, IL-17A, and IL-18 promote EMT of AT II, whereas IL-22 inhibits this process. (5) Fibroblasts: IL-6 and IL-25 promote the proliferation of fibroblasts. IL-4, IL-11, IL-13, and IL-25 induce differentiation of fibroblasts, whereas IL-27 suppresses both events. IL-18 contributes to the senescence of fibroblasts, and IL-37 facilitates autophagy of fibroblasts. IL-1β, IL-4, IL-6, IL-11, IL-13, IL-17A, IL-25, and IL-33 promote collagen synthesis, while IL-7, IL-12, and IL-27 inhibit collagen synthesis.

The level and function of interleukins are also altered in animal models of pulmonary fibrosis (Fig. 1 and Table 2). Interleukins can promote inflammation by regulating immune cell aggregation, thereby affecting pulmonary fibrosis. In bleomycin-treated mice, IL-1β (an IL-1 subtype with pro-inflammatory activity), induced and activated by inflammasome in damaged lung tissue, promotes recruitment of neutrophils and lymphocytes, leading to inflammation at the injury site, as well as pulmonary fibrosis^44^. Similarly, IL-5 (a type-2 cytokine) is increased in lung tissue of bleomycin-induced mice and promotes eosinophil recruitment, as well as pulmonary fibrosis^45^.

**Table 2:** Mechanisms of interleukins in pulmonary fibrosis.

Name	Subtype	Pro-/anti-Inflammatory^a^	Pro-/anti-Fibrosis^b^	Mechanisms	Ref.
IL-1α	IL-1 family	+	+	IL-1α promotes the conversion of fibroblasts to a pro-inflammatory phenotype, which secretes inflammatory cytokines.	^66^
IL-1β	IL-1 family	+	+	IL-1β promotes the recruitment of neutrophils and lymphocytes, leading to inflammation at the injury site and pulmonary fibrosis.	^44^
			+	IL-1β stimulates fibroblasts to synthesize collagen and produce fibrin.	^67,68^
IL-4	γc (IL-2) family and type-2 cytokine	/	+	IL-4 stimulates fibroblasts to induce collagen gene expression.	^55,56^
			+	IL-4 promotes the conversion of fibroblasts to myofibroblasts by activating JNK/ERK signaling, reducing COX gene expression in fibroblasts, and inhibiting PGE2 production.	^57,71^
			−	IL-4 inhibits T cell inflammation and limits lung injury.	^58^
IL-5	Type-2 cytokine	/	+	IL-5 promotes lung eosinophil recruitment, which induces the expression of cytokines leading to fibrosis.	^45^
IL-6	IL-6/IL-12 family	±	+	In bleomycin-treated mice, M2-like macrophages induce the formation of the IL-6/sIL-6Ra complex, which stimulates IL-6 *trans-signaling* in lung fibroblasts and other cells to promote ECM production and cell proliferation.	^51,52^
			+	In the bleomycin-induced fibrotic microenvironment, IL-6, secreted by polarized M2-like macrophages, cooperates with IL-4 and IL-13 to promote aggravation of fibrosis in mice.	^53^
			+	IL-6 regulates proliferation and apoptosis of IPF fibroblasts by ERK signaling and STAT3 signaling.	^72–75^
			+	IL-6, secreted by ATIIs in a paracrine way, activates fibroblasts and promotes pulmonary fibrosis through STAT3 signaling.	^88^
			±	IL-6 acts on ATIIs at the early stage of pulmonary fibrosis and macrophages at the late stage, playing an anti-fibrotic and pro-fibrotic role, respectively.	^54^
IL-7	γc (IL-2) family	/	−	IL-7 mediates the increase of Smad7 through JAK/STAT signaling and exerts an anti-fibrotic effect.	^69^
			−	IL-7 was found to inhibit TGF-β-mediated phosphorylation of PKC-δ (protein kinase C-δ) in fibroblasts from fibrotic lung, but not from the normal counterpart.	^70^
IL-8	CXC chemokine family	/	+	IL-8 derived from MPCs promotes proliferation, differentiation, and migration of MPCs in an autocrine manner, and also induces macrophage migration to the fibroblastic foci through receptors CXCR1/2.	^95^
IL-9	γc (IL-2) family and type-2 cytokine	±	−	IL-9 exerts an anti-inflammatory activity and a protective role in bleomycin-induced pulmonary fibrosis.	^61^
			−	Overexpression of IL-9 promotes PGE2 production of macrophages to inhibit silica-induced pulmonary fibrosis.	^62^
			+	Neutralization of IL-9 by a specific Ab reduces silica-induced lung inflammation and fibrosis in mice.	^63^
IL-10	IL-10 family	−	−	IL-10 inhibits downregulation of IFN-γ and upregulation of TGF-β1 by IFN-γ signaling in bleomycin-induced mice, thereby delaying the process of pulmonary fibrosis.	^46^
			+	Long-term overexpression of IL-10 can promote fibrosis by activating M2 macrophages.	^86,87^
IL-11	IL-6/IL-12 family	/	+	IL-11 promotes fibrin synthesis and fibrosis via the transduction of non-classical ERK signal in an autocrine way.	^78^
			+	IL-11 stimulates fibroblast phenotype transformation and promotes collagen synthesis regulated by ERK kinase in vitro.	^79^
IL-12	IL-6/IL-12 family and type-1 cytokine	/	−	IL-12 may induce transformation of Th2 cells to Th1 cells, thereby upregulating IFN-γ expression, inhibiting collagen production by fibroblasts to suppress fibrosis.	^96^
IL-13	Type-2 cytokine	/	+	IL-13 significantly increases the expression of α-SMA and collagen I in IPF lung fibroblasts, while it has no remarkable effect on normal fibroblasts.	^76^
			+	IL-13 induces differentiation of fibroblasts to myofibroblasts via multiple mechanisms: (1) Regulating the JNK signal. (2) Promoting fibroblast proliferation by inhibiting COX expression and PGE2 production. (3) Promoting differentiation of fibroblasts via upregulating YY1 (Yin Yang 1) expression in AKT signaling.	^57,71,77^
IL-17A	IL-17 family	+	+	IL-17A induces an immunosuppressive microenvironment and suppresses activation of autophagy in the fibrotic lung tissue.	^47^
			+	IL-17A induces EMT in A549 cells by activating the classical Smad2/3 pathway and non-classical ERK1/2 pathway.	^91^
IL-18	IL-1 family	+	+	IL-18 promotes lung fibrosis by downregulating the anti-senescence protein Klotho in lung fibroblasts.	^48^
			+	IL-18 induces EMT by upregulating α-SMA, transcription factor Snail-1, and downregulating E-cadherin, thus participating in the development of bleomycin-induced pulmonary fibrosis.	^49^
IL-22	IL-10 family	−	−	IL-22 exerts an anti-fibrotic effect via inhibiting EMT by targeting alveolar epithelia.	^92,93^
IL-23	IL-6/IL-12 family	/	+	IL-23 mediates the production of IL-17 by γδ T cells or CD4^+^ T cells to promote pulmonary fibrosis.	^98,99^
IL-25 (IL-17E)	IL-17 family and type-2 cytokine	/	+	IL-25 is secreted by AECs and can promote proliferation, differentiation, and collagen synthesis of fibroblasts by binding to IL-17BR.	^80,81^
			+	IL-25 induces ILC2 to release IL-13, which promotes fibrosis.	^100^
IL-27	IL-6/IL-12 family	−	−	IL-27 inhibits fibroblast proliferation and differentiation, collagen synthesis, as well as TIMP1 expression, whereas promotes secretion of MMP2 and MMP9.	^83^
			−	IL-27 regulates the differentiation of T cells, affecting the release of other cytokines against lung fibrosis.	^101^
IL-32γ	Not classified	+	−	Recombinant IL-32γ exerts an anti-fibrotic effect by inhibiting the integrin-mediated activation of FAK/paxillin, a critical pathway in fibroblast activation.	^84^
IL-33	IL-1 family	/	+	Polarization of M1 macrophages to M2 macrophages, promoted by IL-33 via IL-33/ST2 signal, is one of the pathological features in IPF.	^85^
IL-37	IL-1 family	−	−	IL-37 plays an anti-fibrotic effect via promoting the expression of autophagy activation marker LC3II and inducing autophagy in fibroblasts.	^50^

*ATIIs* type II alveolar cells, *COX* cyclooxygenase, *ECM* extracellular matrix, *EMT* epithelial-mesenchymal transition, *ERK* extracellular signal-regulated kinase, *ILC2* type 2 congenital lymphocytes; *JNK* c-Jun N-terminal kinase, *MMP* matrix metalloproteinase, *MPCs* mesenchymal progenitor cells, *PGE2*: prostaglandin E2, *PKC-δ* protein kinase C-δ, *TGF-β1* transforming growth factor-β1, *TIMP* tissue inhibitor of metalloproteinase, *YY1*: Yin Yang 1, *α-SMA* α-smooth muscle actin.

^a^Pro-inflammatory: +; anti-inflammatory: −; dual role: ±; neither or unknown: /.

^b^Pro-fibrosis: +; anti-fibrosis: −; dual role: ±.

Altered interleukin levels in animals with pulmonary fibrosis could impact their weight, survival rate, etc. As an anti-inflammatory and anti-fibrotic cytokine, IL-10 inhibits the downregulation of IFN-γ and upregulation of TGF-β1 in bleomycin-induced pulmonary fibrosis mice, thereby reducing the number of infiltrated inflammatory cells and development of lung fibrosis. Overexpression of IL-10 reduces weight loss and survival rate drop in bleomycin-instillation mice^46^. IL-17A, a pro-inflammatory cytokine also known as IL-17, inhibits the activation of autophagy and autophagy-related cell death in bleomycin-induced lung injury to promote fibrosis. Intravenous anti-IL-17A neutralizing antibody increased survival of bleomycin-injured mice^47^.

Interleukins influence pulmonary fibrosis mainly through inflammation and immune response, but also via other ways. In bleomycin mice models, IL-18, a pro-inflammatory cytokine, induces the senescence of lung fibroblasts by downregulating the anti-senescence protein Klotho^48^. Besides, IL-18 induces EMT by upregulating α-SMA, transcription factor Snail-1, and downregulating E-cadherin, thus participating in the development of bleomycin-induced pulmonary fibrosis^49^. After bleomycin treatment, intranasal instillation of IL-37 (an anti-inflammatory interleukin) plays an anti-fibrotic effect via promoting the expression of autophagy activation marker LC3II and inducing autophagy in fibroblasts. But, regrettably, expression of IL-37 decreases in the lung tissue of IPF patients and mouse models^50^.

IL-6 (acts as both a pro-inflammatory and an anti-inflammatory cytokine) is a member of the IL-6/IL-12 cytokine family. In bleomycin-treated mice, M2-like macrophages induce the formation of the IL-6/sIL-6Ra complex, which stimulates IL-6 *trans-signaling* in lung fibroblasts and other cells to promote ECM production and cell proliferation^51,52^. In the bleomycin-induced fibrotic microenvironment, M2 macrophages polarize and secrete IL-6, which, together with IL-4 and IL-13 (both are type-2 cytokines produced by Th2 cells), activates M2-like macrophages possessing hyper-profibrotic phenotype, then the hyper-profibrotic macrophages accumulate and finally induce extracellular matrix deposition and aggravate pulmonary fibrosis^53^. Also, the elevated IL-6 level may be related to decreased lung function^52^.

The above studies suggested that interleukins exacerbate or alleviate pulmonary fibrosis via a variety of ways. And some interleukins may serve a dual role in pulmonary fibrosis. For example, IL-6 may act on ATIIs and is anti-fibrotic in the early stage, but act on fibroblasts, as well as macrophages and play a pro-fibrotic role in the late stage^54^. IL-4 was initially thought to be pro-fibrotic and then was believed to have no effect on pulmonary fibrosis, while Huaux et al. reported that it has different roles between early and late stages, similar to IL-6 ^56–60^. The role of IL-4 in pulmonary fibrosis may be controversial and needs further investigation.

IL-9 is a secreted protein that belongs to the γc family and type-2 cytokines. The role of IL-9 in pulmonary fibrosis is also controversial. Arras et al. found that IL-9 exerts an anti-inflammatory activity and a protective role in bleomycin-induced pulmonary fibrosis^61^. Furthermore, overexpression of IL-9 promotes PGE2 production of macrophages to inhibit silica-induced pulmonary fibrosis^62^. However, Sugimoto et al. showed that neutralization of IL-9 by a specific Ab reduces silica-induced lung inflammation and fibrosis in mice^63^.

## Main target cells of interleukins in pulmonary fibrosis

### Fibroblasts

Fibroblasts play an essential role in the progression of pulmonary fibrosis. Cytokines induce excessive secretion of collagen from fibroblasts and promote the differentiation from fibroblasts to myofibroblasts^64,65^. Recently, evidence suggested that interleukins directly interact with fibroblasts to promote or inhibit pulmonary fibrosis.

In vitro study has demonstrated that IL-1α (a pro-inflammatory subtype of the IL-1 family) secreted by the alveolar epithelia under stress directly promotes the formation of pro-inflammatory phenotypes of fibroblasts, which further secrete other cytokines to promote pulmonary fibrosis^66^. IL-1β, another IL-1 subtype released by lung macrophages, stimulates fibroblasts to synthesize collagen and produce fibrin^67,68^.

A study demonstrates that IL-37, an anti-inflammatory interleukin, decreases collagen deposition by fibroblasts and alleviates pulmonary fibrosis via inhibiting TGF-β signal transduction. In addition, IL-37 also promotes the autophagy of fibroblasts and regulates cell proliferation, as well as metabolism by inhibiting PI3K/AKT, ERK, and MAPK signaling pathways to protect against fibrosis^50^.

IL-7, a member of the γc family, can induce the synthesis of inflammatory mediators by monocytes and also has anti-fibrotic effects. It appears to have different sensitivities to abnormal and normal fibroblasts. In pulmonary fibrosis, TGF-β signaling induces fibroblast activation and collagen synthesis, while Smad7 can block this process by inhibiting TGF-β signaling. IL-7 mediates the increase of Smad7 through JAK/STAT signaling and exerts an anti-fibrotic effect. Especially, IL-7 only works this way in fibroblasts from IPF patients, not from healthy subjects^69^. Besides, IL-7 was found to inhibit TGF-β-mediated phosphorylation of PKC-δ (protein kinase C-δ) in fibroblasts from fibrotic lung, but not from the normal counterpart^70^.

IL-4 may play a dual role in pulmonary fibrosis. On the one hand, it induces the gene expression of collagen in lung fibroblasts and promotes the differentiation of fibroblasts to myofibroblasts via activation of the JNK/ERK pathway in a time-dependent and dose-dependent manner. The differentiation is also related to the reduction of COX gene expression in fibroblasts and the inhibition of PGE2 production^71^. On the other hand, IL-4 inhibits T cell inflammation and limits lung injury^58^.

As mentioned above, IL-6 plays different roles via acting on different cells at different stages. Also, IL-6 plays opposing roles between fibroblasts from normal subjects and IPF patients. IL-6 is anti-proliferative in normal lung fibroblasts, whereas is strongly pro-proliferative in IPF fibroblasts^72^. In addition, IL-6 upregulates the expression of pro-apoptotic protein Bax and promotes Fas-induced apoptosis through the STAT-3 signaling pathway in normal fibroblasts, while it induces the expression of anti-apoptotic molecule Bcl-2 and proliferation of IPF fibroblasts by ERK signaling pathway^73^. Also, IL-6 promotes the proliferation of IPF lung fibroblasts via the IL-6/STAT3 axis and *trans*-signaling^51,74,75^.

Similar to IL-6, IL-13 has different sensitivities to fibroblasts from normal people and IPF patients. Murray et al. found that IL-13 significantly promotes the expression of α-SMA and collagen I in IPF lung fibroblasts, while the normal fibroblasts do not respond to IL-13 ^76^. Moreover, IL-13 induces differentiation of fibroblasts to myofibroblasts via multiple mechanisms: (1) Regulating the JNK signal. (2) Promoting fibroblast proliferation by inhibiting COX expression and PGE2 production. (3) Promoting differentiation of fibroblasts via upregulating YY1 (Yin Yang 1) expression in AKT signaling^57,71,77^.

IL-11, a member of the IL-6/IL-12 family, can be secreted by multiple cells. Studies have shown that human primary fibroblasts specifically express IL-11 and its receptor IL-11RA. IL-11 promotes fibrin synthesis and fibrosis via transduction of non-classical ERK signal in an autocrine way^78^. In addition, IL-11 stimulates fibroblast phenotype transformation and promotes collagen synthesis regulated by ERK kinase in vitro, thereby promoting the development of pulmonary fibrosis^79^.

IL-25, also known as IL-17E because of the homology with IL-17 family members, is a type-2 cytokine and pro-fibrotic factor. It is secreted by AECs and can promote proliferation, differentiation, and collagen synthesis of fibroblasts by binding to IL-17BR^80,81^.

IL-27, a heterodimeric cytokine that belongs to the IL-6/IL-12 family, is generally believed to be anti-fibrotic^82^. It inhibits the proliferation and differentiation of fibroblasts via inactivating JAK/STAT and TGF-β1/Smad signaling. In addition, IL-27 promotes fibroblasts to secrete MMP2 (matrix metalloproteinase 2) and MMP9, as well as inhibits the expression of TIMP1 (tissue inhibitor of metalloproteinase 1) to resist pulmonary fibrosis^83^.

IL-32 can induce the production of several pro-inflammatory mediators. Hong et al. showed that recombinant IL-32γ exerts an anti-fibrotic effect by inhibiting integrin-mediated activation of FAK/paxillin, a critical pathway in fibroblast activation^84^.

### Macrophages

Under the effect of cytokines, macrophages are involved in the inflammatory response and fibrotic diseases by changing cell phenotypes^30^. Studies have shown that IL-8, IL-33, and IL-10 contribute to pulmonary fibrosis by targeting macrophages.

Macrophages infiltration occurs in IPF lungs. Macrophages migrate to lesion regions under the action of chemokines. Polarization of M1 macrophages to M2 macrophages, promoted by IL-33 via IL-33/ST2 signal^85^, is one of the pathological features in IPF. Besides, long-term overexpression of IL-10 (considered to be anti-fibrotic) can promote fibrosis by activating M2 macrophages^86,87^.

### Alveolar epithelia

Alveolar epithelial are critical cells in the pathogenesis of pulmonary fibrosis. Therefore, cytokines that affect the repair or apoptosis of alveolar epithelia have a definite effect on the development of pulmonary fibrosis. The activated Wnt/β-catenin signaling pathway induces type II alveolar cells (ATIIs) to secrete IL-1β, which enhances TGF-β signaling and promotes the release of IL-6 ^88^. IL-1β and IL-6 induce EMT to promote fibrosis via TGF-β signaling and STAT3 signaling, respectively^88,89^. IL-1β also induces epithelial wound repair^90^.

In bleomycin mice models, endogenous IL-6 regulates ATIIs through STAT3/Akt signaling in an autocrine or paracrine manner. It originates from ATIIs at the inflammatory stage after bleomycin administration. Blocking IL-6 at this stage accelerates pulmonary fibrosis, possibly by enhancing apoptosis of ATIIs^54^.

In vitro evidence suggested that IL-18 and IL-17A both induce EMT^49,91^. Also, in pulmonary fibrosis, IL-22 (an anti-inflammatory cytokine of the IL-10 family) exerts an anti-fibrotic effect via inhibiting EMT by targeting alveolar epithelia, which is the only target cell type of IL-22 in the lung^92,93^.

### Other types of cells

In addition to the target cells described above, other types of cells can also be targeted by interleukins and affect the progression of pulmonary fibrosis^94^. For example, IL-8, secreted by mesenchymal progenitor cells (MPCs), promotes MPCs of IPF lungs to proliferate, differentiate and migrate in an autocrine manner. Besides, IL-8 stimulates macrophages to migrate to the fibroblastic foci through receptors CXCR1/2^95^.

IL-12 (a type-1 cytokine) may induce the transformation of Th2 cells to Th1 cells, thereby upregulating IFN-γ expression, inhibiting collagen production by fibroblasts to suppress fibrosis^96^. IL-33 from fibroblasts and innate immune cells was found to increase IL-13 production by Th2 cells, macrophages, and type II congenital lymphocytes (ILC2) to promote lung fibrosis^97^. Besides, in vivo or in vitro studies demonstrated that IL-25 targets ILC2, while IL-23 and IL-27 (members of the IL-6/IL-12 family) target T lymphocytes to regulate pulmonary fibrosis^98–101^. The specific mechanisms are listed in Table 2.

## Conclusion and future perspectives

In vivo and in vitro studies have shown that altered interleukin levels participate in the formation and development of pulmonary fibrosis by regulating inflammation, immune response, autophagy, senescence, EMT, etc. (Fig. 1). And the target cells of interleukins are mainly fibroblasts, macrophages, and epithelial cells (Table 3).

**Table 3:** Target cells of interleukins in pulmonary fibrosis.

Target cell	Interleukins
Fibroblasts	IL-1α, IL-1β, IL-4, IL-6, IL-7, IL-11, IL-13, IL-18, IL-25, IL-27, IL-32γ, IL-37
Mesenchymal stem cells	IL-8
Alveolar epithelial cells	IL-1β, IL-6, IL-17A, IL-18, IL-22
Macrophages	IL-4, IL-6, IL-8, IL-10, IL-13 Il-33
T cells	IL-12, IL-23, IL-27, IL-33
ILC2s	IL-25

Most interleukins exert either anti-fibrotic or pro-fibrotic effects, whereas few show a dual role or are still controversial as we mentioned above. The sources, signal pathways, and target cells of interleukins, as well as their stability and metabolism in pulmonary fibrosis, need to be clarified. Also, it is important to keep the experimental conditions consistent with previous studies, so that the results and conclusions are comparable. Moreover, some studies were using interleukin overexpressing transgenic mice to investigate the role of a particular interleukin in pulmonary fibrosis, however, the extreme abundance of an individual factor will affect the entire cytokine network and immune regulation, the results from such models may therefore not totally reflect the real state.

Despite the effects of interleukin-targeted treatment on experimental pulmonary fibrosis, clinical applications are lacking and unsatisfactory. Phase II clinical trials (NCT01266135; NCT01629667; NCT01872689; NCT02345070) showed that although IL-13 monoclonal antibodies QAX-576, Tralokinumab, and Lebrikizumab had acceptable safety and tolerability, and Romilkimab (SAR156597, a bispecific Ig-G4 antibody that binds and neutralizes both circulating IL-4 and IL-13) appeared to reduce the occurrence of acute exacerbations in IPF patients, none of these drugs reached the expected efficacy^102–104^. These clinical trials suggest that intervening in one type of interleukins with similar functions in IPF may not be enough to stop the development of fibrosis as it involves a complex network of regulation mechanisms. Intervening interleukins combined with other existing therapy (add-on trial) or targeting interleukins affecting multiple cells/with different functions at the same time may be one of the future directions. For example, therapies that inducing IL-22 production to promote lung epithelial regeneration and using IL-12 to inhibit collagen production by fibroblasts may be able to help improve pulmonary fibrosis. VEGF, FGF, and PDGF signaling pathways or Wnt/β-catenin signaling pathways may be targeted in synergy with ILs. Furthermore, the intervention time is critical as some interleukins play different roles at different stages. These should be tested in experimental animal models first before going to the clinical setting.
